# Supplementary material for: Quantitative proteomic landscape of unstable atherosclerosis identifies molecular signatures and therapeutic targets for plaque stabilization
Source: Commun Biol. 2023 Mar 13;6:265. doi: 10.1038/s42003-023-04641-4 (PMC10011552; doi:10.1038/s42003-023-04641-4)
Supplement: Supplementary file 2 — Supplementary Information [file 42003_2023_4641_MOESM2_ESM.pdf]

Quantitative Proteomic Landscape of Unstable Atherosclerosis Identifies Molecular Signatures and Therapeutic Targets for Plaque Stabilization

Yung-Chih Chen[1,2,3], Meaghan Smith[1], Ya-Lan Ying[1,2], Manousos Makridakis[4], Jonathan Noonan[1,2,3], Peter Kanellakis[1], Alin Rai[3,5,9], Agus Salim[6], Andrew Murphy[2,3,7], Alex Bobik[1,8], Antonia Vlahou[4], David W. Greening[2,3,5,9*], Karlheinz Peter[1,2,3,9*]

*corresponding authors

[1]Atherothrombosis and Vascular Biology Program, Baker Heart and Diabetes Institute, Melbourne VIC, Australia

[2]Central Clinical School, Monash University, Melbourne VIC, Australia

[3]Department of Cardiometabolic Health, University of Melbourne, Melbourne VIC, Australia

[4]Proteomics Research Unit, Biotechnology Division, Biomedical Research Foundation of the Academy of Athens, Athens, Greece

[5]Molecular Proteomics Laboratory, Baker Heart and Diabetes Institute, Melbourne VIC, Australia

[6]Department of Bioinformatics, Baker Heart and Diabetes Institute, Melbourne VIC, Australia

[7]Haematopoiseis and Leukocyte Biology Laboratory, Baker Heart and Diabetes Institute, Melbourne VIC, Australia

[8]Department of Immunology, Monash University, Centre for Inflammatory Disease, School of Clinical Sciences, Monash Health, Melbourne Australia

[9]Department of Cardiovascular Research, Translation and Implementation, La Trobe University, Melbourne VIC, Australia

Corresponding authors: David Greening, PhD, and Karlheinz Peter, MD PhD, Baker Heart and Diabetes Institute, 75 Commercial Road, Melbourne VIC 3004, Australia David.Greening@baker.edu.au; Karlheinz.Peter@baker.edu.au

Short title: Proteome landscape differentiates atherosclerotic plaque instability

Supplementary Figures and Figure Legends

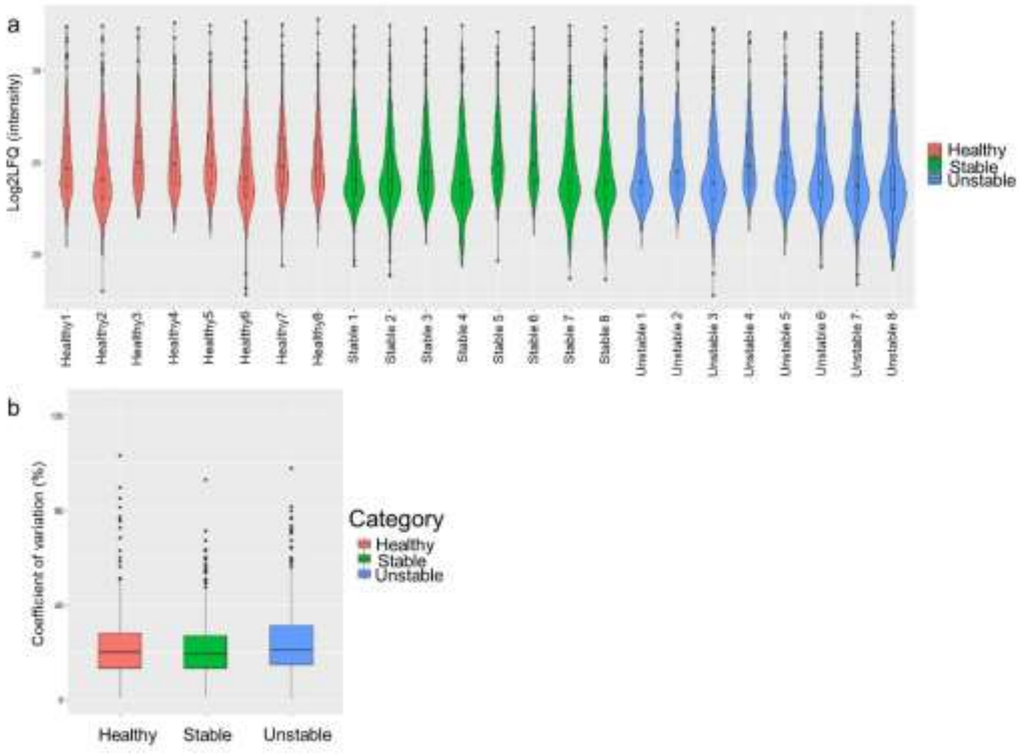

Supplementary Figure 1: (a) Distribution of protein expression across healthy arteries, stable and unstable plaques (n=8). The violin plot depicts the distribution of peptide intensities for each group. (b) The quantified proteome's coefficient of variation. Lower and upper quartiles are shown by boxes, with the median represented by a horizontal line in the center; min and max are represented by whiskers.

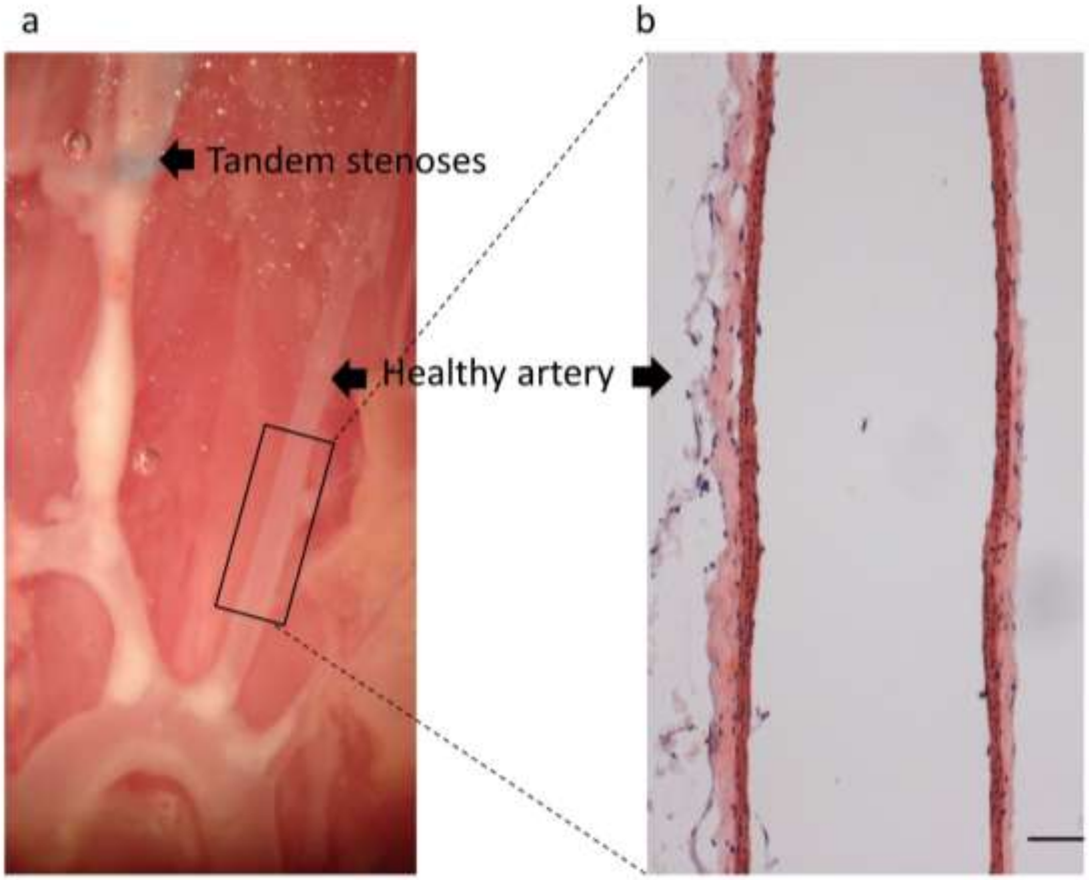

Supplementary Figure 2: Gross anatomy 7 weeks after tandem stenosis surgery in ApoE$^{-/-}$ mice. (a) The blue structure is a coated braided polyester suture. The white material represents atherosclerotic plaques. A longitudinal section of the healthy carotid artery is also provided in H/E staining (b). The bar represents 50 μm.

S100A8

UniProtKB ID P27005 (S10A8_MOUSE)

P27005 (95%), 10,294.8 Da
Protein S100-A8 OS=Mus musculus OX=10090 GN=S100a8 PE=1 SV=3
1 exclusive unique peptides, 2 exclusive unique spectra, 26 total spectra, 24/89 amino acids (27% coverage)

M P S E L E K A L S N L I D V Y H N Y S N I Q G N H H A L Y K N D F K K M V T T E C P Q F V Q N I N I E N L F R E L D I N S D N A I N F E E F L A M V I K V G V
A S H K D S H K E

(K)ALSNLIDVYHNYSNIQGNHHALYK(N)

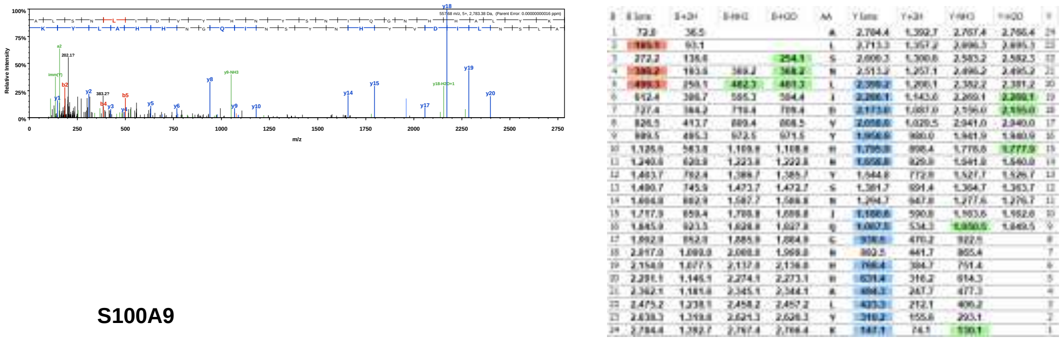

S100A9

UniProtKB ID P31725 (S10A9_MOUSE)

P31725 (100%), 13,049.2 Da
Protein S100-A9 OS=Mus musculus OX=10090 GN=S100a9 PE=1 SV=3
2 exclusive unique peptides, 3 exclusive unique spectra, 32 total spectra, 24/113 amino acids (21% coverage)

M A N K A P S Q M E R S I T T I I D T F H Q Y S R K E G H P D T L S K K E F R Q M V E A Q L A T F M K K E K R N E A L I N D I M E D L D T N Q D N Q L S F E E C
M M L M A K L I F A C H E K L H E N N P R G H G H S H G K G C G K

(R)SITTIIDTFHQYSR(K)

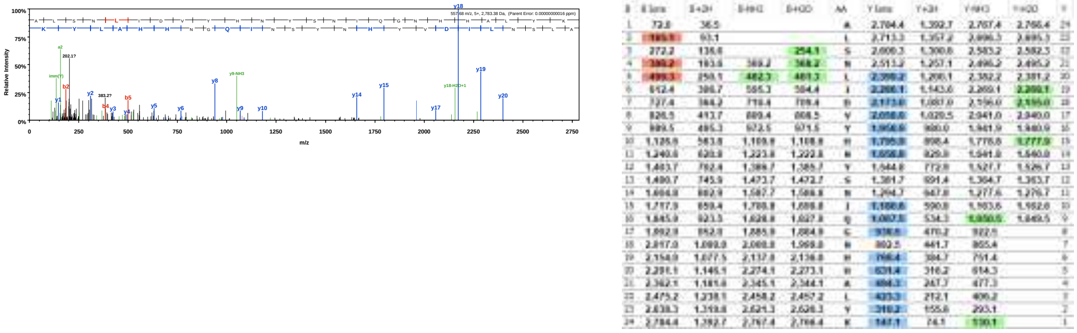

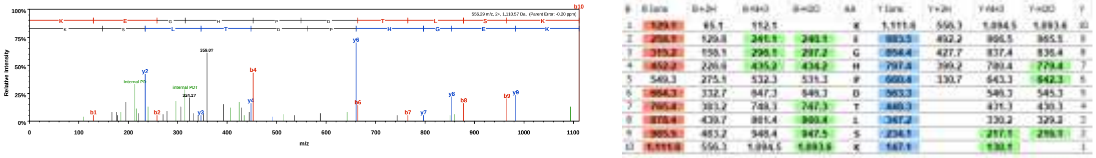

Supplementary Figure 3: Tandem mass spectrometry (MS/MS) spectral output for S100A8 and S100A9 peptides (UniProtKB ID P27005 (S10A8_MOUSE), P31725 (S10A9_MOUSE)) in unstable plaque proteomes. Peptide spectral profiles identified are displayed for each unique peptide sequence (highlighted yellow, relative to protein sequence), with corresponding B-

and Y-ion series for corresponding matched spectra. The fragment peaks include N-terminal (e.g., b-ions) or C-terminal (e.g., y-ions).

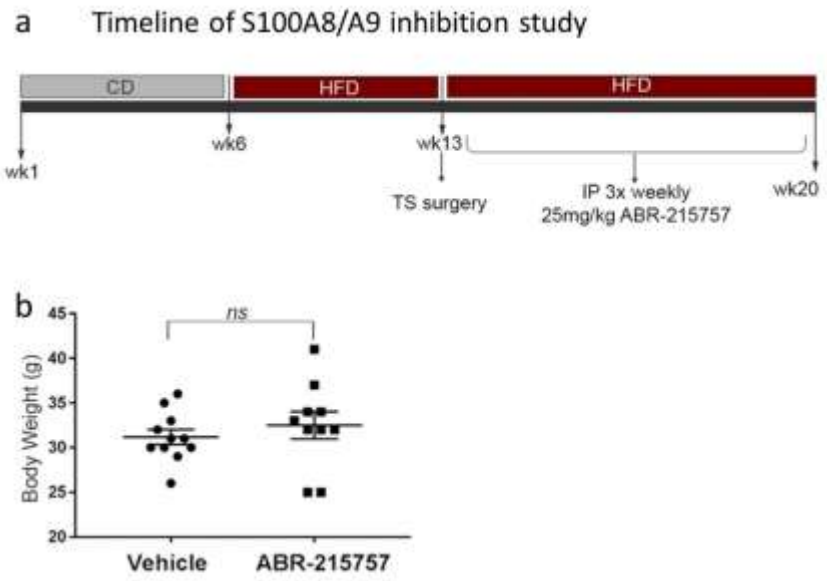

Supplementary Figure 4: S100A8/A9 inhibition study: Design and body weight. (a) Experimental timeline for the ABR-215757 intervention study. (b) Body weight was recorded at the end of the experiment. CD: Chow diet, HFD: High-fat diet (21% fat, 0.15% cholesterol, SF00-219, specialty feeds, Australia).

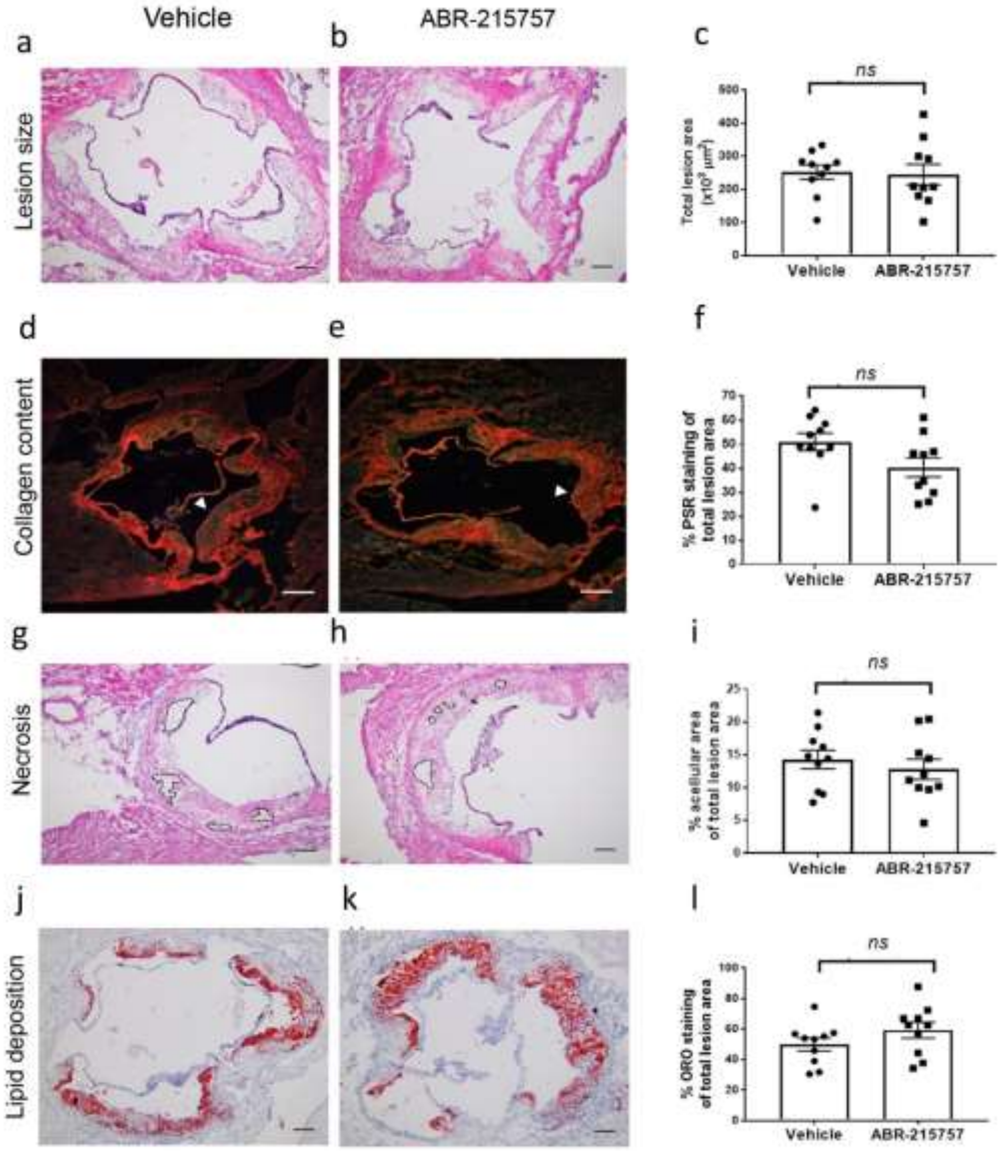

Supplementary Figure 5: No effects of S100A8/A9 inhibition on stable atherosclerosis. (a - c) ABR-215757 had no effect on lesion size in the aortic roots. (d - f) There were no differences in the collagen content as shown by Picro sirus red staining. (g - i) The levels of necrotic core and lipid content (j - l) were determined to be the same. CD: Chow diet, HFD: High-fat diet (21% fat, 0.15% cholesterol, SF00-219, specialty feeds, Australia).

Data represent mean ± S.E.M. Bars represent 25 µm.

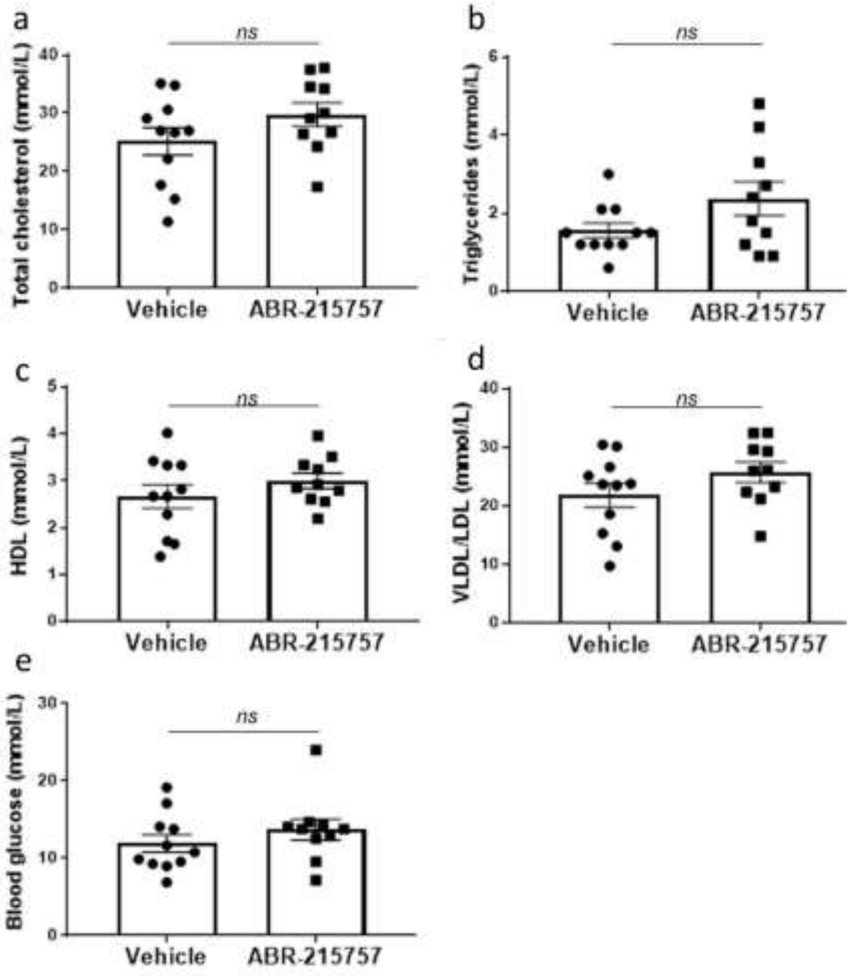

Supplementary Figure 6: At study end point, there were no differences in (a) plasma total cholesterol, (b) triglycerides, (c) HDL, (d) VLDL/LDL, and (e) blood glucose. Mean ± S.E.M. are provided.

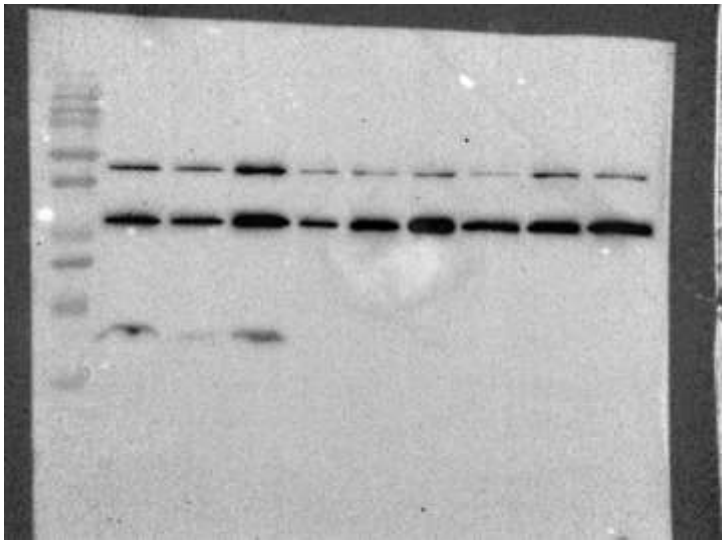

Supplementary Figure 7: Uncropped gel of Figure 4d.
