# Supplementary material for: Quantitative proteomic landscape of unstable atherosclerosis identifies molecular signatures and therapeutic targets for plaque stabilization
Source: Commun Biol. 2023 Mar 13;6:265. doi: 10.1038/s42003-023-04641-4 (PMC10011552; doi:10.1038/s42003-023-04641-4)
Supplement: Supplementary file 3 — Description of Additional Supplementary Files [file 42003_2023_4641_MOESM3_ESM.pdf]

Description of Additional Supplementary Files

File name: Supplementary Data 1
Description: Proteome analysis of healthy, stable, and unstable plaques from TS model.

File name: Supplementary Data 2
Description: Distribution of proteins identified in each region from TS model.

File name: Supplementary Data 3
Description: Differential expression analysis of disease (stable and unstable plaques combined) in comparison to healthy vessels, Reactome pathway enrichment analysis.

File name: Supplementary Data 4
Description: Reactome pathway analysis of healthy aortic region proteome (from Supp Table 2).

File name: Supplementary Data 5
Description: Differential and uniquely identified proteins of stable and unstable mouse aortic extracts.

File name: Supplementary Data 6
Description: Reactome enrichment analysis of differentially expressed proteins in stable and unstable plaque proteomes.

File name: Supplementary Data 7
Description: Comparative analysis of TS model proteome with mouse aortic extract and human ruptured proteome.

File name: Supplementary Data 8
Description: Reactome pathway analysis of stable and unstable plaque proteome with proteins commonly identified in human plaque (from Fig 3D).

File name: Supplementary Data 9
Description: Immunofluorescence antibodies for murine TS plaque protein.

File name: Supplementary Data 10
Description: Immunohistochemistry antibodies and serum for ABR-215757 atherosclerosis study.

File name: Supplementary Data 11
Description: Immunohistochemistry antibodies for human endarterectomy plaque protein validation.
