# Supplementary material for: Quantitative proteomic landscape of unstable atherosclerosis identifies molecular signatures and therapeutic targets for plaque stabilization
Source: Commun Biol. 2023 Mar 13;6:265. doi: 10.1038/s42003-023-04641-4 (PMC10011552; doi:10.1038/s42003-023-04641-4)
Supplement: Supplementary file 5 — Reporting Summary [file 42003_2023_4641_MOESM5_ESM.pdf]

nature portfolio

Corresponding author(s): Karlheinz Peter, David Greening

Last updated by author(s): Jan 12, 2023

Reporting Summary

Nature Portfolio wishes to improve the reproducibility of the work that we publish. This form provides structure for consistency and transparency in reporting. For further information on Nature Portfolio policies, see our Editorial Policies and the Editorial Policy Checklist.

Statistics

For all statistical analyses, confirm that the following items are present in the figure legend, table legend, main text, or Methods section.

n/a	Confirmed	
☐	☒	The exact sample size (*n*) for each experimental group/condition, given as a discrete number and unit of measurement
☐	☒	A statement on whether measurements were taken from distinct samples or whether the same sample was measured repeatedly
☐	☒	The statistical test(s) used AND whether they are one- or two-sided<br>*Only common tests should be described solely by name; describe more complex techniques in the Methods section.*
☐	☒	A description of all covariates tested
☐	☒	A description of any assumptions or corrections, such as tests of normality and adjustment for multiple comparisons
☐	☒	A full description of the statistical parameters including central tendency (e.g. means) or other basic estimates (e.g. regression coefficient) AND variation (e.g. standard deviation) or associated estimates of uncertainty (e.g. confidence intervals)
☐	☒	For null hypothesis testing, the test statistic (e.g. *F*, *t*, *r*) with confidence intervals, effect sizes, degrees of freedom and *P* value noted<br>*Give P values as exact values whenever suitable.*
☐	☒	For Bayesian analysis, information on the choice of priors and Markov chain Monte Carlo settings
☐	☒	For hierarchical and complex designs, identification of the appropriate level for tests and full reporting of outcomes
☐	☒	Estimates of effect sizes (e.g. Cohen's *d*, Pearson's *r*), indicating how they were calculated

Our web collection on statistics for biologists contains articles on many of the points above.

Software and code

Policy information about availability of computer code

Data collection	NA - no custom software or code used
Data analysis	NA - no custom software or code used

For manuscripts utilizing custom algorithms or software that are central to the research but not yet described in published literature, software must be made available to editors and reviewers. We strongly encourage code deposition in a community repository (e.g. GitHub). See the Nature Portfolio guidelines for submitting code & software for further information.

Data

Policy information about availability of data

All manuscripts must include a data availability statement. This statement should provide the following information, where applicable:

- Accession codes, unique identifiers, or web links for publicly available datasets
- A description of any restrictions on data availability
- For clinical datasets or third party data, please ensure that the statement adheres to our policy

Proteomic data (RAW and processed/search files) for each tissue region (healthy, stable, unstable) and comparisons between mouse tissue regions were uploaded to the Proteome Xchange Consortium via the PRIDE partner repository with the dataset identifier PXD030857.
Reviewer account details:
Username: reviewer30165@ebi.ac.uk

Password: WWHIKVLT

Tandem mass spectra were searched as a single batch against the Mus musculus reference proteome (UniProt; UP000000589, 59,345 entries, Feb-2019; canonical protein sequence) supplemented with common contaminants

Statistical analyses were performed using Perseus, R programming, and GraphPad Prism, with unpaired two-sample Student's t-test or one-way ANOVA performed (statistical significance defined at FDR<0.05). Unsupervised hierarchical cluster analysis (unpaired Student's t-test, FDR<0.05). Pathway enrichment map analysis was performed using Cytoscape (v3.7.1), Reactome, and DAVID functional annotation software; significance p<0.05. Unique proteome profile compositions for each vessel segment were graphically visualized using Venny software.15 Protein–protein interaction networks were described using StringApp incorporated into Cytoscape (v3.7.1).

Human research participants

Policy information about studies involving human research participants and Sex and Gender in Research.

Reporting on sex and gender

Use the terms sex (biological attribute) and gender (shaped by social and cultural circumstances) carefully in order to avoid confusing both terms. Indicate if findings apply to only one sex or gender; describe whether sex and gender were considered in study design whether sex and/or gender was determined based on self-reporting or assigned and methods used. Provide in the source data disaggregated sex and gender data where this information has been collected, and consent has been obtained for sharing of individual-level data; provide overall numbers in this Reporting Summary. Please state if this information has not been collected. Report sex- and gender-based analyses where performed, justify reasons for lack of sex- and gender-based analysis.

Population characteristics

Describe the covariate-relevant population characteristics of the human research participants (e.g. age, genotypic information, past and current diagnosis and treatment categories). If you filled out the behavioural & social sciences study design questions and have nothing to add here, write "See above."

Recruitment

Describe how participants were recruited. Outline any potential self-selection bias or other biases that may be present and how these are likely to impact results.

Ethics oversight

Identify the organization(s) that approved the study protocol.

Note that full information on the approval of the study protocol must also be provided in the manuscript.

Field-specific reporting

Please select the one below that is the best fit for your research. If you are not sure, read the appropriate sections before making your selection.

☒ Life sciences ☐ Behavioural & social sciences ☐ Ecological, evolutionary & environmental sciences

For a reference copy of the document with all sections, see nature.com/documents/nr-reporting-summary-flat.pdf

Life sciences study design

All studies must disclose on these points even when the disclosure is negative.

Sample size

Describe how sample size was determined, detailing any statistical methods used to predetermine sample size OR if no sample-size calculation was performed, describe how sample sizes were chosen and provide a rationale for why these sample sizes are sufficient.

Data exclusions

All data exclusions are outlined in the Methods/Results.

Replication

Studies were performed on multiple cohorts of mice at different ages.

Randomization

Describe how samples/organisms/participants were allocated into experimental groups. If allocation was not random, describe how covariates were controlled OR if this is not relevant to your study, explain why.

Blinding

Describe whether the investigators were blinded to group allocation during data collection and/or analysis. If blinding was not possible, describe why OR explain why blinding was not relevant to your study.

Reporting for specific materials, systems and methods

We require information from authors about some types of materials, experimental systems and methods used in many studies. Here, indicate whether each material, system or method listed is relevant to your study. If you are not sure if a list item applies to your research, read the appropriate section before selecting a response.

Materials & experimental systems

n/a	Involved in the study
☐	☒ Antibodies
☒	☐ Eukaryotic cell lines
☒	☐ Palaeontology and archaeology
☐	☒ Animals and other organisms
☒	☐ Clinical data
☒	☐ Dual use research of concern

Methods

n/a	Involved in the study
☒	☐ ChIP-seq
☒	☐ Flow cytometry
☒	☐ MRI-based neuroimaging

Antibodies

Antibodies used

Details of all antibodies are provided in Methods section and complete details within Supplementary Tables 9-11 .
Supplementary Table 9 – Immunofluorescence antibodies for murine TS plaque protein.
Supplementary Table 10 – Immunohistochemistry antibodies and serum for ABR-215757 atherosclerosis study.
Supplementary Table 11 – Immunohistochemistry antibodies for human endarterectomy plaque protein validation.

Validation

i have included only as an example below

1) UCPl- data sheet from manufacturer: suitable for western blot, mouse samples, and validated with brown adipose tissue lysate. 2) α tubulin- data sheet from manufacturer: suitable for western blot, mouse samples, α Tubulin Antibody detects endogenous levels of total α tubulin protein, and does not cross-react with recombinant β tubulin. 3 & 4) pAkt and tAkt- data sheets from manufacturer: suitable for western blot, mouse samples, validated with inhibitors of the PI3K-Akt pathway or Akt siRNA. Previously validated by us in hearts from IGFlR and PI3K mice, and IGFl injection in the current study. 5) Cre Recombinase - data sheets from manufacturer: suitable for western blot, mouse samples. Specificity/ Sensitivity: antibody recognizes transfected and transgenic levels of total Cre recombinase protein. Confirmed with mock-transfected (-) or transfected with a construct expressing Cre recombinase (+). 6) ERalpha- data sheet from manufacturer: suitable for western blot, mouse samples. We included mouse uterus, which has high E alpha, on our western blots as a positive control.

Animals and other research organisms

Policy information about studies involving animals; ARRIVE guidelines recommended for reporting animal research, and Sex and Gender in Research

Laboratory animals	Species, strain and sex have been included.
Wild animals	NA
Reporting on sex	yes
Field-collected samples	NA
Ethics oversight	Alfred Research Alliance Animal Ethics Committee, VIC, AUSTRALIA

Note that full information on the approval of the study protocol must also be provided in the manuscript.
